# Effect of Metals, Metalloids and Metallic Nanoparticles on Microalgae Growth and Industrial Product Biosynthesis: A Review

**DOI:** 10.3390/ijms161023929

**Published:** 2015-10-09

**Authors:** Krystian Miazek, Waldemar Iwanek, Claire Remacle, Aurore Richel, Dorothee Goffin

**Affiliations:** 1 AgricultureIsLife Platform, University of Liege-Gembloux Agro-Bio Tech, Passage des Déportés 2, Gembloux B-5030, Belgium; 2 Faculty of Mathematics and Natural Sciences, the Jan Kochanowski University in Kielce, Swietokrzyska 15, Kielce 25-406, Poland; E-Mail: iwanek@pu.kielce.pl; 3 Genetics and Physiology of Microalgae, Institute of Botany, University of Liege, B22, 27, Bld du Rectorat, Liège B-4000, Belgium; E-Mail: c.remacle@ulg.ac.be; 4 Unit of Biological and Industrial Chemistry, University of Liege-Gembloux Agro-Bio Tech, Passage des Déportés 2, Gembloux B-5030, Belgium; E-Mail: a.richel@ulg.ac.be; 5 Cellule Innovation et Créativité, University of Liege-Gembloux Agro-Bio Tech, Passage des Déportés 2, Gembloux B-5030, Belgium; E-Mail: dorothee.goffin@ulg.ac.be

**Keywords:** microalgae, metal stress, industrial products, growth rate, metal resistance

## Abstract

Microalgae are a source of numerous compounds that can be used in many branches of industry. Synthesis of such compounds in microalgal cells can be amplified under stress conditions. Exposure to various metals can be one of methods applied to induce cell stress and synthesis of target products in microalgae cultures. In this review, the potential of producing diverse biocompounds (pigments, lipids, exopolymers, peptides, phytohormones, arsenoorganics, nanoparticles) from microalgae cultures upon exposure to various metals, is evaluated. Additionally, different methods to alter microalgae response towards metals and metal stress are described. Finally, possibilities to sustain high growth rates and productivity of microalgal cultures in the presence of metals are discussed.

## 1. Introduction

Microalgae are photosynthetic microorganisms, using solar light to convert CO_2_ from the atmosphere into organic carbon. There are eukaryotic microalgae such as green microalgae [1], red microalgae [2], diatoms [3] and dinoflagellates [4] or prokaryotic cyanobacteria [5]. Some of them are capable of growing mixotrophically or heterotrophically because they use sugars, glycerol or organic acids as their carbon source [6]. The optimal temperature for microalgae growth is usually 20–30 °C, but it is also reported that some strains are able to grow at much lower [7] or higher [8] temperature conditions. Microalgae are a source of valuable compounds such as lipids, pigments, carbohydrates, vitamins, and proteins, with potential applications in many branches of industry. Nowadays, research is focused on improving synthesis and maximizing production of valuable compounds from microalgae cultures. Microalgal cells are able to synthetize numerous compounds in higher amounts, as a response to stress conditions such as high temperature, high salinity, nutrient starvation, and also metal stress. However, stress conditions can also have negative effects on microalgae growth [9,10].

Human activity, development of industry and natural Earth processess lead to release of numerous metals (Fe, Zn, Cu, Cd, Cr, Ni, Hg, Pb, La, Li, V), metalloids (As, Te) and metallic nanoparticles (Ag, Pt, TiO_2_, ZnO, CeO_2_, NiO, BaTiO_3_, Y_2_O_3_, Al_2_O_3_) [11,12,13,14,15,16] that can act as stressors or modulators for microalgae growth and metabolism. This review presents advantages and disadvantages of metal stress, as a possible method to produce industrial compounds from microalgae cultures.

## 2. Effect of Metals on Microalgae: Growth Inhibition *vs.* Growth Enhancement

Metals at small concentrations are indispensable for microalgae cells to perform cellular functions. They act as components for photosynthetic electron transport proteins (Cu, Fe) and photosynthetic water oxidizing centres (Mn) or are constituents of vitamins (Co) [17]. They also serve as cofactors for enzymes participating in CO_2_ fixation (Zn in carbonic anhydrase) [18], DNA transcription (Zn in RNA polymerase) and phosphorus acquisition (Zn in alkaline phosphatase) [19] or N_2_ assimilation (Mo, Fe, V in nitrogenase) [20] and nitrate reduction (Mo in nitrate and Fe in nitrite reductase) [21]. However, high concentrations of these metals, and other non-essential heavy metals (Hg, As, Cd, Pb, Cr) cause negative effects (impairment of photosynthetic mechanism, blockage of cell division, inhibition of enzyme activity) in microalgae cells [12]. Metals also influence the morphology of microalgal cells. Accumulation of cadmium (Cd) in *Chlamydomonas acidophila* cells resulted in the increase in cell size and decomposition of polyphosphate bodies [22]. The presence of lead (Pb) in *Chlorella sorokiniana* culture resulted in the formation of colonies of *Chlorella* cells possessing cytoplasm lipid droplets and misshaped chloroplasts [23]. Fragmentation of thylakoid membranes was observed in *Synechocystis* sp. cells upon exposure to thallium (Tl) [24]. Mitochondria in *Desmidium swartzii* cells became enlarged and bloated, upon cell exposure to Zn [25]. Synergistic effect of aluminum (Al) and lead on *Dunaliella tertiolecta* caused cell membrane lysis [26]. Cerium (Ce)-associated cell damage in *Anabaena flosaquae*, can additionally lead to the release of toxins [27]. Lithium (Li) can alter the length and form of flagella in *Chlamydomonas reinhardtii* [28] or affect the structure of polysaccharide sheath around *Ankistrodesmus gracilis* cells [29], and can also at various concentrations inhibit other microalgae strains [30,31]. Cultivation of diatom *Synedra acus* in the presence of germanium (Ge), titanium (Ti), zirconium (Zr) or tin (Sn) caused alterations in shape, size and mechanical strength of silica valves in *Synedra* frustules [32].

Although heavy metals generally have negative effect on microalgae cultures, some reports suggest also their positive role during microalgae cultivation (Table 1). Lead, aluminum [26] and cobalt [33] at low concentrations had stimulatory effect on growth of *Dunaliella tertiolecta* [26] and *Monoraphidium* *minutum* [33]. Arsenic (As(V)) was reported to improve the growth of cyanobacterium *Nostoc minutum* [34] and microalgae *Chlorella salina* [35] and *Chlorella* sp. [36]. What is more, inorganics can support microalgae growth in case of nutrient deficiency. For instance, 20 µg/L vanadium (VO_3_^−^) increased growth of *Scenedesmus obliquus* grown in iron (Fe^3+^) deficient medium up to six times. Vanadium was almost entirely consumed by *Scenedesmus* cells under photoautotrophic cultivation conditions [37]. In another study, addition of 0.01–1 µg/L vanadium (VO_3_^−^) resulted in up to 67% growth enhancement in photoautotrophic *Chlorella pyrenoidosa* culture, even with iron (Fe^3+^) supplementation in the growth media [38]. However, vanadium (VO_3_^−^) at concentrations above 1 mg/L was inhibitory for *Chlorella pyrenoidosa* [38]. Vanadium, in a form of VO_4_^3−^ [39] and V_2_O_5_ [40], was also reported to be inhibitory to *Haematococcus lacustris* [39] and *Scenedesmus quadricauda* [40].

Furthermore, elements from the lanthanide group such as lanthanum (La), cerium (Ce), neodynium (Nd), europium (Eu) or gadolinium (Gd) were reported to constitute a good replacement for calcium deficiency in *Desmodesmus quadricauda* culture, with Gd, La or Nd supplementation leading to nearly the same culture dry weight when compared to Ca supplemented media. Moreover, addition of cerium at low concentration to standard medium increased *Desmodesmus* cell number in culture. However, lanthanide elements increased growth suppression of *Desmodesmus*, when added into manganese deficient medium [41]. Also lanthanum at higher concentration inhibited growth of *Scenedesmus quadricauda* [42] or *Sceletonema costatum* [43], and inhibitory concentration of La was the same as for other lanthanides: cerium (Ce), neodymium (Nd), samarium (Sm), europium (Eu), gadolinium (Gd), terbium (Tb), dysprosium (Dy), holmium (Ho), erbium (Er), thulium (Tm), ytterbium (Yb) and lutetium (Lu) [43]. Cerium (Ce) was stimulatory at lower concentration and inhibitory at higher concentration towards cyanobacterium *Anabaena flosaquae* [27].

Cd^2+^ at small concentrations was reported to stimulate growth and maintain activity of carbonic anhydrase in *Thalassiosira weissflogii* cells, cultivated in Zn-limited medium [44]. Recently, a novel carbonic anhydrase naturally possesing Cd^2+^ as a catalytic metal ion, has been discovered in *Thalassiosira weissflogii* [45].

Ni^2+^ is an essential metal for cultivation of marine diatoms such as *Phaeodactylum tricornutum* [46], *Cyclotella cryptica* [47], *Thalassiosira weissflogii* and *Thalassiosira pseudonana* [48], in the presence of urea as a sole nitrogen source. Nickel serves as a cofactor in an enzyme urease, but Ni at higher concentations was inhibitory for diatom growth [47,48]. A lack of Ni can be partially substituted by cobalt [46].

In addition to metals and metalloids, also metallic nanoparticles (NPs) exert activity towards microalgae. Inhibitory effects of TiO_2_, ZnO, CeO_2_, NiO, BaTiO_3_, Y_2_O_3_, Al_2_O_3_, Ag and Pt nanoparticles were reported towards numerous freshwater and marine microalgae strains and their inhibitory activity was suggested to be due to Reactive Oxygen Species (ROS) generation [49,50] or mechanical damage caused by nanoparticles themselves [51], but also due to metal ions released from nanoparticles [50,52,53], light shading effect [54], interactions with growth media components [55] or simultaneous effect of various factors [56]. Inhibitory activity of nanoparticles also depends on their size [49] and aged suspension [55] or growth medium composition [53]. On the other hand, metal ions released from nanoparticles can also stimulate growth of cyanobacteria and microalgae [57].

**Table 1:** Effect of metals, metalloids and metallic nanoparticles on growth of microalgae.

Metal	Microalgae Strain	Cultivation Time	Concentration	Effect on Growth	Ref.
Hg	*Chlorella* sp. *Scenedesmus acutus*	8 days	2.5–5 mg/L	100% growth inhibition	[58]
Hg	*Selenastrum capricornutum*	–	0.027 mg/L	50% inhibition	[59]
Pb	*Phaeocystis antarctica*	10 days	0.57 mg/L	50% inhibition	[60]
Pb	*Dunaliella tertiolecta*	48 h	1.5–6.4 mg/L	20% stimulation	[26]
		48 h	7.29 mg/L	25% inhibition	
Cr(III)	*Dyctiosphaerium chlorelloides*	72 h	13–17 mg/L	50% inhibition	[61]
Cr(III)	*Scenedesmus* sp.	9 days	0.75 µM	MMC	[62]
	*Geitlerinema* sp.	9 days	0.25 µM		
Cr(VI)	*Chlorella pyrenoidosa*	72 h	2 mg/L	50% inhibition	[63]
Cr(VI)	*Chlorella vulgaris*	96 h	5 µmol/L	~40% inhibition	[64]
As(III)	*Chlorella* sp.	72 h	25.2 mg/L	50% inhibition	[65]
	*Monoraphidium arcuatum*	72 h	14.6 mg/L	50% inhibition	
As(III)	*Chlorella* sp.	72 h	27 mg/L	50% inhibition	[66]
As(V)	*Chlorella* sp.	72 h	1.1 mg/L	50% inhibition	[66]
As(V)	*Chlorella* sp.	72 h	25.4 mg/L	50% inhibition	[65]
	*Monoraphidium arcuatum*	72 h	0.254 mg/L	50% inhibition	
As(V)	*Oscillatoria tenuisa*	72 h	3.8 mg/L	50% inhibition	[67]
	*Anabaena affinis*	72 h	2.6 mg/L	50% inhibition	
	*Microcystis aeruginosa*	72 h	1.2 mg/L	50% inhibition	
As(III)	*Nostoc minutum*	7 days	5 mg/L	Cell death	[34]
As(V)	*Nostoc minutum*	7 days	1000 mg/L	66% stimulation	[34]
Cu	*Isochrysis galbana*	72 h	0.01–0.018 mg/L ^T^	50% inhibition	[68]
Cu	*Phaeocystis antarctica*	10 days	0.0059 mg/L	50% inhibition	[60]
Cd	*Phaeocystis antarctica*	10 days	1.5 mg/L	50% inhibition	[60]
Cd	*Scenedesmus armatus*	24 h	~15–18 mg/L ^+^ or 0.46–0.54 mg/L ^+x^	50% inhibition	[69]
Cd	*Thalassiosira weissflogii*	–	4.6 pM	~30%–92% stimulation ^ZnL^	[44]
Ni	*Selenastrum capricornutum*	–	0.125 mg/L	50% inhibition	[59]
Ni	*Synechococcus* sp.	15 day	25 mg/L	~42% inhibition	[70]
Li	*Chlorella vannielii*	12 h	1000 mg/L	48% inhibition	[30]
Li	*Cyanothece* sp.	28 days	70 mg/L	Cell death	[31]
Tl	*Chlorella* sp.	72 h	80 nmol	100% inhibition	[71]
Tl	*Synechocystis* sp.	72 h	1 µM	50% inhibition	[72]
Co	*Monoraphidium minutum*	11 days	0.5 ppm	12% stimulation	[33]
			3 ppm	44% inhibition	
Zn	*Phaeocystis antarctica*	10 days	1.11 mg/L	50% inhibition	[60]
Zn	*Anabaena* sp.	96 h	0.38 mg/L	50% inhibition	[73]
Al	*Dunaliella tertiolecta*	48 h	2.6–14.9 mg/L	20% stimulation	[26]
		48 h	22.42 mg/L	25% inhibition	
Al	*Isochrysis galbana*	72 h	2.57–3.23 mg/L ^T^	50% inhibition	[68]
V ^Met^	*Scenedesmus obliquus*	7 days	20 µg/L	534% stimulation *	[37]
V ^Met^	*Chlorella pyrenoidosa*	7 days	1 µg/L	67% stimulation	[38]
V ^Met^	*Chlorella pyrenoidosa*	7 days	>1 mg/L	Inhibitory threshold	[38]
V ^Ort^	*Haematococcus lacustris*	4 days	2.5–5 mM	Full inhibition	[39]
V ^Oxi^	*Scenedesmus quadricauda*	12 days	2.23 mg/L	50% inhibition	[40]
Ce	*Desmodesmus quadricauda*	3 days	6 µmol/L	16% stimulation *^A^*	[41]
Ce	*Desmodesmus quadricauda*	3 days	94 µmol/L	~19% inhibition *^A^*	[41]
Ce	*Desmodesmus quadricauda*	3 days	5.74 µmol/L	20% inhibition *^B^*	[41]
				60% stimulation *^C^*	
Ce	*Desmodesmus quadricauda*	3 days	1.14 µmol/L	40% inhibition *^D^*	[41]
Ce	*Anabaena flosaquae*	17 days	0.1 mg/L	~16% stimulation	[27]
			5–10 mg/L	~33% inhibition	
La	*Desmodesmus quadricauda*	3 days	5.72 µmol/L	10% inhibition *^B^*	[41]
				80% stimulation *^C^*	
La	*Desmodesmus quadricauda*	3 days	1.13 µmol/L	No change *^D^*	[41]
La	*Scenedesmus quadricauda*	22–23 days	72 µmol/L	50% inhibition	[42]
La, Ce, Nd, Sm, Eu, Gd, Tb, Dy, Ho, Er, Tm, Yb, Lu	*Skeletonema costatum*	96 h	28–29 µmol/L	50% inhibition	[43]
Nd	*Desmodesmus quadricauda*	3 days	5.76 µmol/L	10% stimulation *^B^*	[41]
				120% stimulation *^C^*	
Nd	*Desmodesmus quadricauda*	3 days	1.09 µmol/L	~5% inhibition *^D^*	[41]
TiO_2_-NPs	*Nitzschia closterium*	96 h	88–118 mg/L	50% inhibition	[49]
TiO_2_-NPs	*Pseudokirchneriella subcapitata*	72 h	2.53 mg/L	50% inhibition	[52]
TiO_2_-NPs	*Chlorella vulgaris*	–	2.5–5 g/L	42% inhibition	[74]
ZnO-NPs	*Chlorella vulgaris*	72 h	200 mg/L	35% cell viability	[50]
ZnO-NPs	*Dunaliella tertiolecta*	96 h	2.4 mg/L	50% inhibition	[56]
ZnO-NPs	*Pseudokirchneriella subcapitata*	72 h	0.1 mg/L	80% inhibition	[52]
ZnO-NPs	*Phaeodactylum tricornutum*	–	100 mg/L	80% inhibition	[51]
	*Alexandrium minutum*		100 mg/L	80% inhibition	
	*Tetraselmis suecica*		100 mg/L	No effect	
ZnO-NPs	*Scenedesmus rubescens*	96 h	14.27 mg/L or >810 mg/L ^CM^	50% inhibition	[53]
CeO_2_-NPs	*Pseudokirchneriella subcapitata*	72 h	4.1–6.2 mg/L ^AS^	50% inhibition	[55]
NiO-NPs	*Chlorella vulgaris*	120 h	44 mg/L	50% inhibition	[75]
Y_2_O_3_-NPs	*Phaeodactylum tricornutum*	–	100 mg/L	~40% inhibition	[51]
	*Alexandrium minutum*		100 mg/L	~40% inhibition	
	*Tetraselmis suecica*		100 mg/L	70% inhibition	
BaTiO_3_-NPs	*Chlorella vulgaris*	72 h	1 mg/L	~57% inhibition	[76]
Al_2_O_3_-NPs	*Chlorella* sp.	72 h	45.4 mg/L	50% inhibition	[54]
	*Scenedesmus* sp.	72 h	39.35 mg/L	50% inhibition	
Ag-NPs	*Pseudokirchneriella subcapitata*	72 h	1.63 mg/L	50% inhibition	[77]
Pt-NPs	*Pseudokirchneriella subcapitata*	72 h	16.9 mg/L	50% inhibition	[77]
nZVI-Nanofer 25	*Arthrospira maxima*	216 h	5.1 mg/L	19% stimulation	[57]
nZVI-Nanofer 25	*Desmodesmus subspicatus*	216 h	5.1 mg/L	73% stimulation	[57]
nZVI-Nanofer 25	*Parachlorella kessleri*	216 h	5.1 mg/L	38% stimulation	[57]

MMC, Minimum Metal Concentration significantly affecting Chlorophyll a intensity; ^T^, depending on temperature applied; ^+^, depending on Cd salt used; ^x^, including complex abilities of media mineral elements; *, when compared to *Scenedesmus* growth in Fe deficient medium; ^ZnL^, at low Zn concentrations; ^Met^, added as metavanadate; ^Ort^, added as orthovanadate; ^Oxi^, added as vanadium pentoxide; *^A^*, in standard medium and compared to a control in standard medium without Ce; *^B^*, in Ca deficient medium and compared to a control in standard medium without tested metal; *^C^*, in Ca deficient medium and compared to a control in Ca deficient medium without tested metal; *^D^*, in Mn deficient medium and compared to a control in Mn deficient medium without tested metal; NPs, nanoparticles; ^CM^, depending on culture medium; ^AS^, depending on aged suspension; nZVI, zero-valent iron nanoparticles; Ref., Reference.

## 3. Metal Stress as a Method for Stimulation of Bioproduct Synthesis

Accumulation of metals in microalgae cells consists of two mechanisms: metal adsorption on the cell wall surface containing functional groups (carboxyl, hydroxyl, phosphate, amino, sulfhydryl) and absorption of metals inside cells via metal transport systems [12,19,78]. Metals in microalgae cells can cause formation of reactive oxygen species (ROS) such as hydroxyl radical (·OH), superoxide anion (O_2_·^−^), singlet oxygen (O_2_*) and hydrogen peroxide (H_2_O_2_) that interact with lipids, proteins and nucleic acids, resulting in their degradation. As a protective response to metal induced oxidative stress, microalgae cells synthetize chelating agents such as phytochelatin or exopolymers in higher amounts [12,79,80]. Chelating agents are organic compounds that form two or more bonds with a metal ion, thereby creating a coordination complex chelate–metal and preventing metal ions from interaction with biological macromolecules [81]. Another defense mechanism againsts oxidative stress is the synthesis of antioxidant compounds (pigments, glutathione, ascorbate) or enzymes (superoxide dismutase, catalase) that are responsible for quenching reactive oxygen species (ROS) and also reducing metal ions into their less reactive forms [12,79,80]. Therefore, oxidative stress can be considered as a trigger mechanism to induce production of target compounds by metal-exposed microalgae cells, under conditions where the detrimental effect of metals on microalgal culture is avoided.

### 3.1. Pigments

Chlorophylls, carotenoids and phycobilins are microalgal pigments that harvest light in the process of photosynthesis. Chlorophylls are primary photosynthic pigments that contain tetrapyrrole macrocycle rings and are present in various forms (a, b, c1, c2, c3, d, f), in different microalgae or cyanobacteria species (Table 2). Green microalgae possess chlorophyll content up to 6.7% [82], and upon chemical modifications, to phaeophytin [83] or Cu^2+^-chlorophyllin [84], can be used as a biomordant [83] to enchance the dyeing process of textile products or as a textile dye [84] with antimicrobial properties. Additionally, an Mg^2+^ ion in a chlorophyll centre can be substituted with Zn^2+^, Ni^2+^, Cd^2+^, Pb^2+^, Co^2+^ or Pt^2+^ [85,86,87,88,89,90]. Carotenoids–accessory photosynthetic pigments, are fat-soluble tetraterpenoid molecules that are divided into no oxygen-containing carotenes (β-carotene) and oxygen-containing xanthophylls (lutein, astaxanthin, zeaxanthin) [91]. Phycobiliproteins are water-soluble proteins that serve as accessory pigments in blue-green or red microalgae, giving a blue (c-phycocyanin, allophycocyanin) [34,92] or pink, red (b-phycoerythrin, c-phycoerythrin) [93,94] colour. Chlorophylls, carotenoids and Phycobiliproteins can find applications in food, cosmetic and pharmaceutical products as coloring, antioxidant, food additive or therapeutic agents [95,96,97].

**Table 2:** Types of chlorophyll present in eukaryotic microalgae and cyanobacteria.

Chlorophyll Type	Microalgae Strain	Taxonomy	Reference
a, b	*Chlorella vulgaris*	Green microalgae	[98]
a, c1, c2	*Phaeodactylum tricornutum*	Diatoms	[99]
a, c1, c2	*Kryptoperidinium foliaceum*	Dinoflagellates	[100]
a, c2, c3	*Karenia mikimotoi*	Dinoflagellates	[100]
a, d	*Acaryochloris marina*	Cyanobacteria	[101]
a, f	*Halomicronema hongdechloris*	Cyanobacteria	[102]

The presence of metals can have an enchancing effect on pigment content in microalgae or cyanobacteria cells. Copper (Cu^2+^) at concentration between 0.05–0.2 g/L induced β-carotene production in *Chlamydomonas acidophilla* [103]. The change in iron (Fe^2+^) medium concentation resulted in a growth improvement and an increase in lutein, zeaxanthin and β-carotene content in *Coccomyxa onubensis* cells [104]. Also, β-carotene content in *Dunaliella salina* cells was increased seven times in the presence of 450 µM Fe^2+^ and 67.5 mM acetate, however at the expense of four-fold reduction in *Dunaliella* cell number [105]. Cyanobacterium *Nostoc minutum* cultivated photoautotrophically in medium containg 1 g/L arsenic(V) was reported to posses chlorophyll, carotenoid and allophycocyanin content higher by 75%, 40% and 25%, respectively, when compared to control culture [34]. Similarly, small concentrations of Ni (0.1–10 µM) increased chlorophyll content and c-phycocyanin production even by 47% and up to 4.35 times, respectively, in *Anabaena* *doliolum* culture [92]. The content of c-phycocyanin, phycoerythrin and allophycocyanin in cyanobacterium *Phormidium tenue* culture increased considerably in the presence of As, but the uplift profiles were strongly dependent on As dosage (0.1–100 ppm) and exposure time [106]. In other studies, cultivation of *Synechocystis* sp. in the presence of Pb and Cd, and *Spirulina platensis* in the presence of Pb, showed a decrease in biomass and pigment (chlorophyll, carotenoid, phycocyanin) concentration, in the culture volume. Nevertheless, pigment content in cyanobacteria biomass increased at some metal concentrations and cyanobacteria growth was stimulated at low Pb concentrations [107,108]. Lead (Pb) and cadmium (Cd) at concentrations up to 10 mg/L increased chlorophyll concentration in cultures of metal resistant *Scenedesmus quadricauda* and *Pseudochlorococcum typicum* [109]. Tellurium (TeO_3_^2−^), added into *Spirulina platensis* growth media, was accumulated and incorporated into peptides in *Spirulina* cells. As a result, production of Te-phycocyanin and Te-allophycocyanin possessing enhanced antioxidant activity, was reported in *Spirulina platensis* cells [110].

### 3.2. Lipids

Microalgal cells are a source of lipids including triacyloglycerols (TAGs) and fatty acids [111], but also phytosterols [112] and sphingolipids [113], with potential applications as biofuels, nutraceuticals and food additives. It is reported that nutrient deficiency such as nitrogen deprivation results in oxidative stress and lipid accumulation in microalgal cells [114]. Cultivation of *Chlorella minutissima* in the presence of Cd (0.2–0.4 mM) or Cu (0.2–1 mM) leads to the increase in both biomass density and cell lipid content, providing lipid productivity improved 2.17-fold with 0.4 mM Cd or by 34% with 0.4 mM Cu [115]. *Euglena gracilis* cultivated photoautotrophically or mixotrophically in the presence of low chromium (Cr^6+^) concentration exhibited higher total lipid content, although lipid stimulation (10%–100%) was dependent on *Euglena* strain used and medium composition tested [116]. Addition of 0.1 g/L TiO_2_ nanoparticles with UV-A irradiation applied, slightly increased production of fatty acids in *Chlorella vulgaris* cells, without growth reduction [74]. Recently, zero-valent iron nanoparticles (5.1 mg/L) were reported to increase lipid productivity in *Arthrospira maxima*, *Desmodesmus subspicatus* and *Parachlorella kessleri* cultures, respectively by 40%, 2.75-fold and by 66% [57]. Metal stress also causes the alteration of fatty acid profile in microalgae cells. The effect of As(III) on *Nannochloropsis* sp. cells resulted in a slight increase in cell lipid content and a change in lipid profile, as the decrease in polyunsaturated fatty acids and the increase in short-chain saturated (C16:0, C18:0) and monounsaturated (C16:1, C18:1) fatty acids, was depicted [117]. Nickel at 0.5 mg/L caused a shift of fatty acid profile towards saturated fatty acids (C14:0, C16:0, C20:0) in *Dunaliella salina* and *Nannochloropsis salina* cells, also with the upshift of saturated C18:0 and unsaturated C18:2 for *Nannochloropsis* and C22:0 behenic acid for *Dunaliella* [118]. Composition of fatty acids (chain length, number of double bonds) defines the biodiesels produced from corresponding triglycerides in terms of their quality and properties (including cetane number, density, viscosity, lubricity, calorific value, NO*_x_* emissions) [119,120,121]. Therefore, metal stress can be applied to alter composition of fatty acids in microalgal cells and produce biodiesel of desirable quality and properties [117]. As a contrary, cultivation of *Nannochloropsis limnetica* and *Trachydiscus minutus* in the presence of zero-valent iron nanoparticles (nZVI) caused the decrease in saturated fatty acids (C14:0, C16:0, C18:0) and the increase in eicosapentaenoic acid (C20:5ω3) content in *Nannochloropsis* and *Trachydiscus* biomass [57]. Eicosapentaenoic acid (EPA) can be used as a nutraceutical or pharmacological agent for the treatment of heart and inflammatory diseases [122].

### 3.3. Exopolymers

Extracellular polymeric substances (EPS), consisting of exopolysaccharides and exoproteins, are excreted by microalgae and cyanobacteria upon exposure to stress factors such as nutrient (N, P) imbalance, but the release mechanism can also depend on cultivation conditions (light intensity, temperature, salinity, microelement availability) and the stage of microalgal growth [123,124,125,126,127,128]. Exopolysaccharides can be of linear or branched structure and contain C6 (glucose, galactose, fructose, rhamnose, fucose) and C5 (xylose, arabinose) sugars, as well as uronic (glucuronic, galacturonic) acids, aromatic, pyruvate, acetate, sulphate and halide groups. Additionally, extracellular polysaccharides can be also coupled with peptides, lipids and nucleic acids [129,130].

Metals were reported to stimulate the release of exopolymers by microalgal cells. A considerable increase in the release of exopolysaccharides and extracellullar proteins was observed in the culture of cyanobacterium *Lyngbya putealis*, as a response to the presence of Cu and Co [131]. Increased release of extracellular polymers from *Thalassiosira weissflogii* [132], and *Thalassiosira pseudonana* [133] in the presence of Ag [132] and Cd [133] ions released from engineered nanoparticles (ENPs), was also reported. Extracellular polymeric substances possess antiviral, antitumor, anticoagulant, antiinflammatory and immunostimulant activity, but they can also serve as biosurfactants, biolubricants, bioemulsifiers [130] and a source of sugars for biofuels [134].

### 3.4. Phytochelatin

Phytochelatins are (oligo)peptides synthetized in plants, yeast, algae and cyanobacteria for detoxification of heavy metals. The structure of phytochelatin is (γ-Glu-Cys)_n_-Gly with γ-Glu-Cys *n* being between 2 to 11. Phytochelatin is synthetized by phytochelatin synthase (glutathione-γ-glutamylcysteinyltransferase), by firstly adding γ-Glu-Cys from glutathione (γ-Glu-Cys-Gly) to another glutathione molecule forming (γ-Glu-Cys)_2_-Gly (PC2) and further incorporates new γ-Glu-Cys units into PC2 [135]. Synthesis of short chain phytochelatins (2 to 6 of γ-Glu-Cys units) was reported in cells of microalgae (Table 3) such as *Scenedesmus vacuolatus* [136], *Phaeodactylum tricornutum* [137,138,139], *Scenedesmus armatus* [140], *Stichococcus bacillaris* [141], *Micrasterias denticulata* [142] and cyanobacterium *Anabaena doliolum* [143] exposed to increasing concentration of Cd, Pb, Cu and/or As. Phytochelatin content in *Scenedesmus armatus* and *Stichococcus bacillaris* cells exposed to constant (Const.) concentration of Cd and As respectively can be also further elevated, with the upshift of CO_2_ supplementation for *Scenedesmus* [140] and decrease of pH for *Stichococcus* [141]. Also synthesis of iso-phytochelatins such as Cys(GluCys)*_n_*Gly and (GluCys)*_n_*Ala was reported in *Chlamydomonas reinhardtii* upon Cd exposure [144]. Phytochelatins, obtained from microalgae cultures, can become a component for biosensors, designed for detection of heavy metals in samples of environmental, biological or pharmaceutical origin [145,146].

**Table 3:** Synthesis of phytochelatin in microalgae exposed to heavy metals.

Strain	Metal	Metal Uplift	Phytochelatin Uplift	PCN ^A^	Growth Rate ^C^	Reference
*Scenedesmus vacuolatus*	Cd	0.3→79 nM	~3→25 amol/cell	PC2	Reduced by 37%	[136]
			~1→44 amol/cell	PC3		
			~0→17 amol/cell	PC4		
*Phaeodactylum tricornutum*	Cd	0→0.45 µM	~0.16→3.6 amol/cell	PC2	No change	[137]
			~0.5→1.3 amol/cell	PC3		
			~0.05→1.5 amol/cell	PC4		
*Phaeodactylum tricornutum*	Cu	0.068 pM→0.4 µM	~0.16→1.7 amol/cell	PC2	No change	[137]
			~0.5→1.5 amol/cell	PC3		
			~0.05→0.8 amol/cell	PC4		
*Phaeodactylum tricornutum*	Cd	0→10 µM	~0→12.5 amol/cell	PC2	Toxic effect avoided	[138]
			~0→25 amol/cell	PC4		
			~0→5 amol/cell	PC5		
*Phaeodactylum tricornutum*	Pb	0→10 µM	~0→50 amol/cell	PC2	Toxic effect avoided	[138]
			~0→13 amol/cell	PC3		
			~0→3 amol/cell	PC5		
*Phaeodactylum tricornutum*	Cu	0→10 µM	~2→18 amol/cell	PC2	–	[139]
			~0→38 amol/cell	PC3		
			~0→5 amol/cell	PC6		
*Scenedesmus armatus*	Cd	Const. 93 µM *	~40→200 nmol-SH/g	PC2	Reduced by 26%	[140]
			~80→1300 nmol-SH/g	PC3		
			~20→280 nmol-SH/g	PC4		
*Stichococcus bacillaris*	As(III)	Const. 100 µM **	0.07→0.15 µmol-SH/g	PC2	Reduced by 20%	[141]
	As(V)	Const. 100 µM **	0.14→0.38 µmol-SH/g	PC2	Reduced by 30%	

^A^ Phytochelatin with N number of γGlu-Cys units; ^C^ when compared to control; * increase of CO_2_ supplementation from 0.1% to 2%; ** pH shift from 8.2 to 6.8.

### 3.5. Phytohormones

Zeatin, indoleacetic acid and abscisic acid are phytohormones that can be used as growth regulators for plants [147,148], and yeast [149], but also as anti-aging agents [150] and potential drugs for neural [151] or cancer [152] diseases. Phytohormones can be found in microalgae [153] and their content can be amplified in the presence of heavy metals. The content of indoleacetic acid, zeatin and abscisic acid increased in *Chlorella vulgaris* cells grown in the medium containing 10^−4^ M Cd, Pb or Cu, however at the expense of decreased cell number in the culture. Interestingly, addition of 10^−8^ M brassinolide into metal-containing *Chlorella* culture enabled to achieve cell number comparable to control culture, together with further stimulation of zeatin, indoleacetic acid and abscisic acid production [154].

### 3.6. Organoarsenical Compounds

**Figure 1:**
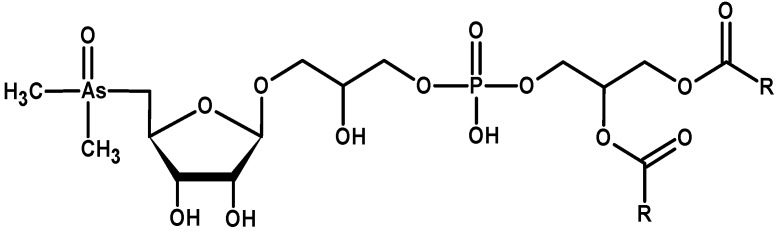
Chemical structure of dimethylarsenoriboside phospholipids (R—a carbon chain of fatty acid).

Accumulation of As in microalgae cells has been recently extensively summarized [155]. In essence, the uptake of As(V) from surroundings into microalgae cells is accomplished by means of phosphate transport system, while As(III) is transported by aquaglyceroporins and hexose permeases [155]. Subsequently, As(V) is reduced to As(III) via As reductase action, with simultaneous oxidation of glutathione (GSH). As(III) undergoes methylation via As methyltransferase action into monomethylarsonate (MMA) and dimethylarsinate (DMA). Arsenic(III) can also undergo bio-oxidation to As(V) or be extruded from cells [156,157,158]. Arsenic(V) can be incorporated into cellular components such as sugars and lipids. In microalgae, dimethylarsinate (or its reduced form: dimethylarsinous acid) can combine with the adenosyl group from *S*-adenosyl methionine, leading to formation of a dimethylarsinyladenosine, which further undergoes glycosidation to dimethylarsenoribosides [159,160]. In cyanobacteria, dimethylarsinate undergoes reduction, ribose-coupling and glycosidation [161]. Some varieties of arsenosugars containing glycerol, sulphate, sulphonate and phosphate groups were detected for microalgae [160,162]. Arsenolipids in microalgae were determined as dimethylarsenoriboside phospholipids (Figure 1), although phospholipids containing single As(V) or DMA groups were also reported [163]. Content and compositions of arsenoorganics formed in microalgae *Chlorella* and *Monoraphidium* [65], *Dunaliella* and *Phaeodactylum* [163], *Chlamydomonas* [160] or cyanobacteria *Synechocystis* [157,161] and *Nostoc* [161] cells depends on microalgae strain used, as well as on arsenic(V) concentration applied, exposure time and phosphate availability. Arsenolipids and arsenosugars are currently evaluated as possible therapeutic agents [164]. However, application of As-containing compounds is limited due to high toxicity and so far, only derivatives of arsenolipids have been reported to possess any medical applications [159].

### 3.7. Nanoparticles and Nano-Needles

Nanoparticles are particles with sizes ranging between 1–100 nm [165]. Nanoparticles possess antiviral, antibacterial, antifungal, anticancer and antiparasite activity. They also find application in the field of catalysis or photonics or can serve as drug carriers and components of chemical sensors [166]. Methods applied for manufacturing nanoparticles range from mechanical, laser and UV irradiation treatment to microemulsion system, hydrothermal process, sol–gel process, chemical vapor condensation, sonochemical treatment and microbial biosynthesis [165,167]. Synthesis of nanoparticles by microorganisms (bacteria, yeast, fungi and microalgae) can constitute a green and environmentally friendly method for nanoparticles production [168,169]. Formation of nanoparticles: Au, Ag or Pd (Table 4) from metal ions solutions takes place inside microalgae cells (intracellularly) or in the media (extracellularly) via interactions with molecules of microalgal cell metabolism (NADH, pigments, peptides, proteins and polysaccharides) [170,171,172,173,174,175,176]. The size of synthetized nanoparticles depends on microalgal strain and metal type used, but place of synthesis, initial metal loading, light and temperature are also crucial factors influencing formation of nanoparticles. Additionally, synthesis of Cd nanoparticles in a form of CdS [177] or Ni nanoparticles as a product of reduction of other nanoparticles (NiO) [75], was also reported. Besides nanoparticles, biosynthesis of nanoneedles by microalgae also occurs; such nanoneedles, composed of zinc and phosphorous, were detected in *Scenedesmus obliquus* cells as a result of exposure to high Zn concentration [178].

**Table 4:** Synthesis of nanoparticles (NP) in microalgae and cyanobacteria cultures.

Element NP	Source	Strain	Place of Synthesis	Average Particle Size (nm)	Reference
Gold (Au)	HAuCl_4_·3H_2_O	*Chlorella vulgaris*	Intracellularly	40–60	[170]
Gold (Au)	KAuCl_4_	*Eolimna minima*	Intracellularly	5–100	[171]
Silver (Ag)	AgNO_3_	*Parachlorella kessleri*	Extracellularly	9, 14 or 18	[172]
Silver (Ag)	AgNO_3_	*Botryococcus braunii*	Extracellularly	15.67	[173]
Silver (Ag)	AgNO_3_	*Scenedesmus* sp.	Intracellularly	15–20	[174]
Palladium (Pd)	Na_2_(PdCl_4_)	*Chlorella vulgaris*	Microalga culture	7	[175]
Palladium (Pd)	PdCl_2_	*Chlorella vulgaris*	Intracellularly	5–12	[170]
Palladium (Pd)	PdCl_2_	*Plectonema boryanum*	Extracellularly	≤30	[176]
Cadmium sulphide (CdS)	Cd(NO_3_)_2_·4H_2_O	*Scenedesmus*	Intracellularly	120–175 (described as nanoparticles)	[177]
Nickel (Ni)	NiO–NPs	*Chlorella vulgaris*	Microalga culture	–	[75]

## 4. Influence of Growth Conditions on Microalgal Resistance Towards Metals

Metals at low concentration can be stimulatory for growth and production of target compounds, but metal overdose has detrimental and lethal effects on microalgae cultures. Hence, microalgal cultivation in metal polluted wastewaters should be designed in such a way to limit cell–metal interactions to the level at which metal concentration exerts only beneficial effects on microalgae growth and biosynthesis of crucial products. Microalgal cell response to metal presence depends on many factors such as conditions of cultivation, nutrient availability, presence of organic compounds and tolerance ability of particular strains.

### 4.1. Growth Media Composition and Cultivation Conditions

Composition of growth media is a crucial factor regarding microalgae response towards heavy metals, such as arsenic, cadmium or nickel.

Arsenate (AsO_4_^3−^) and phosphate (PO_4_^3−^) are mutual competitors for the uptake by microalgal cells [155]. A 10–fold increase in phosphate concentation resulted in a 18 times higher resistance of *Monoraphidium arcuatum* against As (V). On the other hand, a 10-fold decrease in medium nitrate NO_3_^−^ content at ordinary (PO_4_^3−^) concentation, decreased by 28% *Monoraphidium* resistance towards arsenic [65]. In another study, a 131-fold phosphate uplift improved 516 times resistance of *Chlorella salina* against As (V) [35]. Indeed, increasing concentration of As (V) stimulated growth of arsene tolerant *Chlorella* sp. at low phosphate (P) concentration, although cell yields obtained were lower than in experiments with high P concentration [36]. Concentration of PO_4_^3−^ in medium in relation to dissolved lead content can be also important, as Pb^2+^ can precipitate in a form of Pb_3_(PO_4_)_2_, thereby removing available phosphate from solution and inhibiting growth of *Chlamydomonas reinhardtii* [179].

Sulphur is a component of cysteine that participates in the defense mechanisms against heavy metals. The resistance of *Chlamydomonas moewusii* exposed to 4 mg/L cadmium can be improved five times and cysteine cell content can be raised 10 times, when sulphate (SO_4_^2−^) concentration in medium is increased 100 times [180]. In another study, a 10-fold increase in SO_4_^2−^ supply resulted in a *Chlamydomonas reinhardtii* resistance improved by up to 77% towards Cd. Improved *Chlamydomonas* resistance was accompanied with an increased activity of cysteine desulfhydrase, an enzyme responsible for the cleavage of cysteine into pyruvate, NH_3_ and sulfide, the latter one reported to react with Cd to form CdS [181].

A 20-fold increase in ammonium (NH_4_^+^) concentration increased five times the accumulation of PO_4_^3−^ in *Chlorella* *vulgaris* cells and caused a 50% alleviation in inhibition of *Chlorella* growth exerted by chromium (Cr) [182]. Increase in magnesium (Mg^2+^) and hydrogen (H^+^) concentration reduced nickel toxicity towards *Pseudokirchneriella subcapitata*, as Mg^2+^ and H^+^ compete with Ni^2+^ for the uptake by the cell transport system [183]. In other studies, an increase in H^+^ concentration was reported to improve, even up to 23 times [184], *Chlorella* sp. resistance against Cu.

Zn alleviated detrimental effects of Cr on the photosynthetic mechanism in *Micrasterias denticulata* cells and Fe ameliorated inhibitory effect of Cd and Cr on *Micrasterias* cell development. Ca and Gd were reported to prevent alterations in cell morphology caused by Pb and Cd, thereby nullifying negative effects of Pb and Cd on *Micrasterias* cells [185].

Finally, toxicity of thallium towards *Chlorella* sp. was completely alleviated, when concentration of K^+^ in media was increased 20 times, presumably due to competive uptake in *Chlorella* cell transport systems [71].

Cultivation parameters such as light intensity and CO_2_ concentration are also important factors affecting microalgae response towards metals. Alterations in ligh irradiance had influence on inhibition or stimulation of *Chlamydomonas reinhardtii* growth under different Cu concentrations, and also affected accumulation of Cu in *Chlamydomonas* cells [186]. Increase of CO_2_ supply enabled the alleviation of the inhibitory effect of Cd towards *Scenedesmus armatus*, although growth inhibition was not entirely overcome [140].

### 4.2. Supportive Compounds

Another modulating approach could be supplementation of microalgae cultures with organic compounds such as phytohormones or chelating agents.

#### 4.2.1. Phytohormones: Modulating Effect

Phytohormones—spermidine (polyamine), gibberellin and many representatives of auxin and cytokinin groups—were reported to prevent inhibition of *Chlorella vulgaris* culture exposed to cadmium (Cd), copper (Cu) or lead (Pb) at a concentration of 0.1 mM. What is more, addition of compounds from the cytokinin group such as benzyladenine, zeatin, kinetin, 2-isopentenyladenine, diphenylurea, forchlorphenuron and thidiazuron not only enabled restoration of the *Chlorella* culture, but also increased cell number by up to 77%, when compared to control. Supplementation of spermidine, gibberellin, auxins or cytokinins generally increased not only the content of chlorophyll, carotenoid, protein, ascorbate and glutathione in *Chlorella* cells, but also activity of superoxide dismutase and catalase [187]. In earlier studies, it was stated that the inhibitory effect of 0.1 mM Cd, Cu and Pb on *Chlorella vulgaris* culture can be also nullified in the presence of brassinolide [154].

#### 4.2.2. Chelating Agents: Modulating Effect

Chelating agents are synthetized by microalgae for intracellular (phytochelatin, glutathione) or extracellular (exopolymers) detoxification of metals, but can also be added artificially into growth media to bind metals and modulate cell–metal interactions. Such agents can be low-molecular organic acids (ethylenediamine tetraacetic acid, nitrilotriacetic acid, citrate) or humic substances: humic acid or fulvic acid (Table 5).

Addition of 34 µM ethylenediamine tetraacetic acid (EDTA) into *Scenedesmus subspicatus* culture enabled a ~55% reduction in growth inhibition exerted by ~40 µM Cu [188]. Also EDTA, as well as nitrilotriacetic acid (NTA) and citrate (Cit), were reported to prevent accumulation of lanthanum (La), gadolinum (Gd) and yttrium (Y) in *Chlorella vulgaris* cells, with reduction in accumulation around 10- to 30-fold higher for EDTA, when compared to NTA and Cit [189]. On the other hand, citrate was reported to enhance Cd (0.25 µM/L) accumulation and growth inhibition of *Selenastrum capricornutum*, due to the occasional uptake of Cd-citrate by cells [190]. With the absence of EDTA in growth medium, cadmium (Cd) exerted much stronger inhibitory effects on *Scenedesmus armatus*, when compared to the growth in EDTA-containing medium [69]. Growth of *Scenedesmus quadricauda* or *Microcystis aeruginosa* in the presence of lanthanum (0.72–72 µM) and EDTA (0.269–26.9 µM) was inhibited or enhanced, depending on La and EDTA concentrations. EDTA (2.69–13.4 µM) vastly alleviated the inhibitory effect of La on *Microcystis* growth, although EDTA alone and at higher concentration had strong inhibitory effects towards *Microcystis* [42]. EDTA [37,191,192] or citrate [37,193] increased Fe availability to microalgae, although high concentration of chelating agent can have opposite effects [42,191]. Additionally, EDTA that fails to maintain availabilily of Fe at high pH during *Spirulina* cultivation, can be replaced by alternative chelating agents such as Fe complexes of *N*,*Nʹ*-bis(2-hydroxybenzyl)ethylenediamine-*N*,*N'*-diacetic acid (HBED), ethylenediamine-*N*,*N'*-bis((2-hydroxyphenyl)acetic acid) (EDDHA) or ethylenediamine-*N*,*N'*-bis((2-hydroxy-4-methylphenyl)acetic acid) (EDDHMA) [194].

Humic acid was reported to protect *Dunaliella salina* and *Nannochloropsis salina* cells against Ni^2+^ stress, by means of forming humic acid–Ni^2+^ complexes and/or by adsorbing on cell surface and thus, creating an additional barrier for Ni^2+^ uptake [118]. Similarly, humic acids reduced toxicity of Cd^2+^ and Zn^2+^ towards *Pseudokirchneriella subcapitata* [195], Hg^2+^ towards *Isochrysis galbana* [196] and ZnO nanoparticles towards *Anabaena* sp [197]. Humic acid itself at 7 and 2.5–10 mg/L stimulated growth of *Isochrysis galbana* [198] and *Stichococcus bacillaris* [141], presumably due to improved nutrient uptake via humic acid–cell membranes interaction [198]. However, an opposite effect, enhanced toxicity of Pb towards *Isochrysis* in the presence humic acid, was also observed [198], because the formation of a ternary complex between Pb, humic acid and microalga cell surface, enhances internalization of Pb [199]. Humic acid was also reported to be inhibitory (0.3 mg/L) and lethal (1 mg/L) for *Anabaena circinalis*, probably due to its chelating activity towards Fe^3+^, leading to the decrease in availability of Fe necessary for *Anabaena* growth [200]. It is also noteworthy, that humic acid can undergo degradation under high light irradiance, leading to the decreased capacity for metal complexation [201]. Fulvic acid contributed to protection of *Scenedesmus subspicatus* against Cu^2+^ [188], but no protective effect against Cd^2+^ and Zn^2+^ was found for *Pseudokirchneriella subcapitata* [195]. Fulvic acid was also reported to serve as a source of phosphorus to nullify toxic effects of aluminum (Al) on P-metabolism in *Chlorella pyrenoidosa* [202].

**Table 5:** Effect of humic and fulvic acids on microalgae response towards metals.

Chelating Agent	Metal	Uplift of Chelating Agent Concentration	Strain	Reduction of Growth Inhibition	Reference
Humic acid (Soil)	Ni^2+^ (0.5 mg/L)	0→0.2 mg/L	*Dunaliella salina Nannochloropsis salina*	40% ^A^→25% ^C^30% ^A^→15% ^C^	[118]
Humic acid (Soil)	Cd^2+^ (0.2 mg/L)	0→5 mg/L	*Pseudokirchneriella subcapitata*	52% ^A^→28% ^C^	[195]
Humic acid (Soil)	Zn^2+^ (0.39 mg/L)	0→5 mg/L	*Pseudokirchneriella subcapitata*	55% ^A^→4% ^C^	[195]
Humic acid (Peat)	Cd^2+^ (0.2 mg/L)	0→5 mg/L	*Pseudokirchneriella subcapitata*	52% ^A^→8% ^C^	[195]
Humic acid (Peat)	Zn^2+^ (0.39 mg/L)	0→5 mg/L	*Pseudokirchneriella subcapitata*	55% ^A^→30% ^C^	[195]
Humic acid	As(III) (100 µM)	0→10 mg/L	*Stichococcus bacillaris*	52% ^A^→33% ^C^	[141]
Humic acid (Sediment)	Hg^2+^ (10 ppb)	0→10 ppm	*Isochrysis galbana*	Complete reduction in growth inhibition plus stimulation	[196]
Humic acid	ZnO–NPs (1 mg/L)	0→3 mg/L	*Anabaena* sp.	70% ^A^→40% ^C^	[197]
Fulvic acid (Sediment)	Cu^2+^ (~5 µM)	1→5 mg/L	*Scenedesmus subspicatus*	56% ^A1^→30% ^C1^	[188]
Fulvic acid (Suwannee River)	Cd^2+^ (0.2 mg/L)	0→5 mg/L	*Pseudokirchneriella subcapitata*	52% ^A^→45% ^C^	[195]
Fulvic acid (Suwannee River)	Zn^2+^ (0.39 mg/L)	0→5 mg/L	*Pseudokirchneriella subcapitata*	No reduction in growth inhibition	[195]
Fulvic acid (Soil)	Al ^i+o^ (6 µM)	0→11 mg/L	*Chlorella pyrenoidosa*	Complete reduction in growth inhibition plus stimulation	[202]

^A^ growth inhibition in the absence of chelating agent; ^A1^, growth inhibition in the presence of decreased amount of chelating agent; ^C^ growth inhibition in the presence of chelating agent; ^C1^, growth inhibition in the presence of increased amount of chelating agent; ^i+o^, a sum of inorganic and organic aluminum.

#### 4.2.3. Nanoparticles: Modulating Effect

The presence of metallic and non-metallic nanomaterials can alter the effect of metals on microalgae. For instance, the presence of graphene oxide (GO) increased toxicity of Cd towards *Microcystis aeruginosa* [203], while Cd toxicity towards *Chlamydomonas reinhardtii* was reduced in the presence of titanium dioxide engineered nanoparticles (ENPs) [204]. TiO_2_ nanoparticles and Zn ions in the mixture exerted the enhanced or decreased toxicity towards *Anabaena* sp., depending on mutual interactions between different concentrations of TiO_2_ and Zn [73]. Finally, the presence of engineered nanoparticles was reported to decrease intracellular content of Cu and Pb in *Chlorella kesslerii* and wall-possessing *Chlamydomonas reinhardtii*, as metal binding to nanoparticles reduces availability of Cu and Pb to these microalgal strains [205].

#### 4.2.4. Macrocycles: Modulating Effect

Supramolecular water soluble compounds such as cyclodextrins, calixarenes and resorcinarenes can possibly change interactions between microalgae and metals.

Cyclodextrins (CDs) are macrocyclic oligosaccharides composed of six, seven, or eight (α 1–4) glucosidic units and called: α,β and γ-CDs, respectively. They are produced from enzymatic hydrolysis of starch, with cycloglycosyl transferase amylases (CGTases) [206,207]. CDs are ring molecules, either toroidal or cone shaped, but not cylindrical [208]. The primary hydroxyl groups are situated on the narrow side while, the secondary groups are located on the wider side. The central cavity of CDs is hydrophobic, while the outer part is hydrophilic due the presence of hydroxyl groups [209]. β-cyclodextrins can possess methyl, carboxymethyl or hydroxypropyl moieties [210,211] and form complexes with metals [212], phytosterols [213] and carotenoids [214]. Carboxymethyl-β-cyclodextrin (3.3 mM) was successfully harnessed for reduction of metal (Cd, Co, Cu) toxicity towards naphthalene-degrading bacterium *Burkholderia* sp. [215]. On the other hand, alhough hydroxypropyl-β-cyclodextrin up to 20 mM did not itself cause inhibition of microalga *Selenastrum capricornutum* growth, it failed to protect this microalga strain against Zn toxicity [216], because hydroxypropyl-β-cyclodextrin does not possess metal-binding substituents [215].

Calix[*n*]arenes and resorcin[4]arenes are macrocyclic compounds consisting of phenol or resorcinol units, respectively, which are cyclically linked by aliphatic bridges [217]. Calix[*n*]arenes (*n* = 4, 5, 6, 7 and 8) are obtained as a result of condensation of *p*-*tert*-butylphenol with formaldehyde under alkaline catalysis [218,219,220], whereas resorcin[4]arenes are formed as a result of acid-catalysed reaction between resorcinol and aliphatic or aromatic aldehydes [221]. Water-soluble calix[4]arenes and resorcin[4]arenes possess charged groups (ammonium, sulphonium, carboxylate, phosphate) or hydrophilic fragments [222,223,224,225]. Derivatives of calix[*n*]arenes such as *p*-sulphonate or methoxycarboxylic derivatives form stable complexes with Zn^2+^, Cu^2+^, Ni^2+^ under neutral or alkaline conditions [226,227,228]. Water soluble resorcin[4]arene derivatives are able to form complexes, not only with the metal ions, but also with amino acids, sugars, and nucleosides [229,230,231]. It was demonstrated that *p*-sulfonatocalix[4,6,8]arene and *C*-nonylresorcin[4]arene possess antimicrobial activity against fungal and bacterial microorganisms [232]. Additionally, *C*-methylcalix[4]-resorcinarene containing pyridinium salt, was reported to exhibit a selective inhibitory effect on Gram-positive bacteria [233].

Water soluble supramolecular molecules have the potential to modify interactions between metals and microorganisms such as microalgae, but their application in this field is highly unexplored.

### 4.3. Development of Strain Tolerance to Metals

Some microalgae are able to inhabit environments contaminated by heavy metals. Such microalgal strains possess uplifted tolerance towards heavy metals [104,234,235,236,237]. Increased tolerance can be also induced on laboratory scale by applying proper metal dosages [238,239] or metal-containing wastes [240]. It results in development of physiologically adapted strains [61,239,241] or metal resistant mutants due to rare spontaneous mutations that occur before metal treatment [61,238,239]. Microalgae with improved tolerance can become promising microbes for cultivation in metal polluted growth media and for production of target compounds [104]. However, it should be taken into consideration that increased tolerance can be strictly strain–metal specific [235] and a lack of inducing metal in the cultivation medium can have a negative effect on growth of metal resistant mutants [238].

## 5. Strategy for Microalgal Production in the Presence of Metals

It has been widely reported that microalgae cultures, due to their ability for metal accumulation, can be used for bioremediation of heavy metal contaminated water/wastewater streams [80,242,243]. In this review, other aspects of microalgae exposure to metals, such as production of numerous industrially important compounds from metal-exposed microalgae (Table 6) and stategies to alter microalga–metal interactions for industrial microalgae productions, are discussed. As a result of metal exposure, microalgae are able to synthesize a range of target compounds: pigments, lipids, peptides, exopolymers, phytohormones, arsenoorganics or nanomaterials, as a defense mechanism against metal stress. Although metals induce synthesis of compounds by microalgae cells, they may also have detrimental effects on cell number, growth rate, cell dry weight, thereby diminishing productivity of target compounds in a metal-trigger system. For instance, an elevated copper (Cu) concentration increased chlorophyll and carotenoid content in *Dunaliella* cells [244] and stimulated release of polysaccharides from *Cylindrotheca fusiformis* [245] and phenolics from *Dunaliella tertiolecta* [246] cells, though at the expense of a reduced number of cells in the culture. In other studies, the content of chlorophyll, protein and lipids in *Chlorella vulgaris* [247], proline and total amino acids in *Chlorella pyrenoidosa* [63] and chlorophyll and carotenoid in *Pseudokirchneriella subcapitata* [248] increased in the presence of cadmium (Cd), chromium (Cr) and copper (Cu) respectively, but the growth in these cultures was considerably suppressed [63,247,248]. A possible strategy to overcome this problem could be cultivation of microalgae under non-stressed conditions in order to obtain higher cells densities, with subsequent addition of metals for inducing stress and synthesis of target products in microalgae cells [10]. Metals at higher concentration are toxic to microalgae, but at lower concentration can be stimulatory for growth (Table 1). Additionaly, it was concluded that growth media might contain nutrients (Ca, Mg) in amounts that are not sufficient for some microalgal strains to achieve desirable growth [249] and therefore some metal-containing effluents could also serve as a nutrient replacement for Ca [41], Fe [37] or Zn [44] deficiency in growth media. Microalgae cultivation systems require large amounts of water [250] and production of target compounds with metal polluted industrial water streams, instead of exploiting clean water sources, could be an additional advantage. Growth of cyanobacteria *Nostoc linckia* and *Nostoc rivularis* was stimulated at low loadings of (Zn, Cd)-containing sewage waters, but suppressed at high sewage water loadings [251]. Industrial wastes/wastewaters contain not only metals, but also numerous organic pollutants (pesticides, pharmaceuticals, personal care products *etc.*) [252] that can be harmful for microalgae cultures. Furthermore, although metal uptake occurs in microalgal cultures, high dosage wastes can strongly decrease productivity of microalgal cultivation [251,253]. Therefore, precautions should be taken to control concentration of metals and/or organic toxicants, so that optimal microalgal growth and product biosynthesis could be obtained.

An integrated process for metal (Al, Fe, Mn, Ba, Ce, La) remediation and lipid production in cultures of marine microalgae (*Nannochloropsis*, *Pavlova*, *Tetraselmis*, *Chaetoceros*) has already been proposed [254]. Recently, a combination of heavy metal (Zn, Mn, Cd, Cu) removal to increase up to 2.17-fold lipid production from *Chlorella minutissima* has been described [115]. Further, it was concluded that small concentrations of metal mixtures (As, Cd, Co, Cr, Cu, Hg, Ni, Pb, Se, Zn) present in coal fired flue gas could increase lipid yield in *Scenedesmus obliquus* cultures by 61% [255]. It was also suggested that uptake of lead (Pb) from textile dyeing industry effluent by *Neochloris* sp. could be accompanied with accumulation of cell neutral lipid content with increased levels of oleic (C18:1) acid [256]. Additionally, metal exposure can lead to modifications in fatty acid profiles in microalgal cells, thereby improving quality of biodiesel [117]. Finally, the uptake of metals (Cr, Mn, Fe, Co, Ni, Cu, Mo, Cd, Pb) from landfill leachate combined with hydrogen production in *Chlamydomonas reinhardtii* cultures, has been discussed [257]. It should be noted that products, synthesized by microalgae cells in response to metal stress, can be contaminated by metals. The presence of metals in final products might not be appropriate in terms of application for food or medical purposes. Therefore, desorption methods (EDTA, diethyl dithiocarbamate, carbonate, dicarbonate) should be applied to obtain a metal free product, without causing the degradation of the product structure. Moreover, monitoring to maintain metal concentration in a final product below allowable thresholds must be considered.

Microalgae are capable of absorbing heavy metals under photoautotrophic [12,80,242,243] and heterotrophic conditions [234], and hence biocompound production under metal stress possibly could be achieved in open ponds, photobioreactors, but also in fermentation tanks [258]. Strictly controlled media compositions can modulate microalgal sensitivity towards heavy metals also during a chemostat-based continuous cultivation [59]. Additionally, an amount of microalgae biomass in relation to metal concentration should be taken into consideration, as high biomass densities can alleviate detrimental effect of metal ions on microalgae cells in culture [259,260]. The use of older culture inocullum also improved resistance of *Scenedesmus quadricauda* against Ag nanoparticles [239]. Synergistic effects of different heavy metal ions [261] or metal ions with nanoparticles (see Section 4.2.3) on microalgae cells, should be also taken into consideration. Additionally, although nanoparticles can be synthetized by microalgae cells (see Section 3.7), the presence of nanoparticles can have negative effects on microalgae (Table 1).

Composition of growth media and cultivation parameters have significant influence on microalgae resistance towards metal induced stress (see Chapter 4). Moreover, a modification of cultivation media with the change of metal concentration and/or composition can enhance not only growth, but also biosynthesis of target compounds. For instance, an alteration in Fe, Mn, Mo concentration and addition of Ni, caused the increase in biomass and hydrocarbon productivity in *Botryococcus braunii* culture [262]. Also supplementation of growth medium for *Chlorella vulgaris* with 12 µM chelated Fe^3+^, resulted in an increase in *Chlorella* cell number by 27% and lipid content by 625%, when compared to the culture without Fe^3+^ added [263]. In another study, a six-fold uplift in Fe^3+^ concentration enabled an increase of 22% lipid productivity in *Nannochloropsis oculata* culture [264]. *Anabaena variabilis*, cultivated in a new vanadium (VO_3_^−^)-containing growth media, produced 550% more hydrogen, and VO_3_^−^ was suggested as a microelement responsible for amplification of H_2_ synthesis [265]. Addition of 20 µg/L VO_3_^−^ into growth medium increased dry weight by up to 34%, and cell chlorophyll content by up to 100% in heterotrophically cultivated *Scenedesmus obliquus* [37]. Further, 20 µg/L VO_3_^−^ stimulated production of zeaxanthin, lutein and β-carotene in *Chlorella fusca* cultivated at standard Fe medium concentration or Fe deficient conditions, and the stimulatory effect of VO_3_^−^ was more pronounced at standard Fe concentration [266]. Vanadium, added as 1.25 mM Na_3_VO_4_ to *Haematococcus lacustris* culture, increased carotenoid synthesis in cells and carotenoid productivity in culture respectively by 120% and 25%, after a two-day exposure. However, in a prolonged cultivation time, caronenoid productivity decreased drastically if compared to control, presumably due to inhibitory activity of Na_3_VO_4_ towards protein tyrosine phosphatase (PTPase) [39].

Supplementation of organic compounds into microalgal culture can be an additional protection in order to diminish interactions of metals from wastes to a level that enables metal-trigger production of target compounds, together with sufficient microalgal growth rate, even in high metal-level environment. Organic compounds such as phytohormones or various chelating agents inducing resistance mechanisms inside cells or creating a resistance barrier outside cells, can serve as a defense for cultivation of microalgae in high dose-metal contaminated systems. Interestingly, phytohormones can not only protect microalgae against metal stress [154,187], but can also improve growth [267] and increase the content of saturated [268] or unsaturated [267] fatty acids in microalgae cells. Therefore, a proper design of media composition (micro/macro-elements, phytohormones, chelating agents, macrocycles) and cultivation conditions (CO_2_, light, temperature, pH) seems to be necessary in order to avoid detrimental effects of heavy metal ions and to obtain sufficient growth and productivity of target compounds in metal-exposed microalgae cultures. Finally, microalgae strains isolated from heavy metal polluted areas or developed in the laboratory, are able to tolerate increased metal concentrations and can become promising candidates for cultivation under metal stress [104,235,236,240,241]. Such strains are more resistant against detrimental effects of metal exposure and could also be suitable for cultivation and synthesis of target products in outdoor open systems, as metal-stress conditions can prevent contamination by competitive or predatory micro and higher organisms [9,269].

**Table 6:** Some examples of metal effects on microalgae growth and bioproduct synthesis.

Microalgae Strain	Bioproduct	Metal/s	Bioproduct Synthesis *^Info^*	Growth	Reference
		*Pigments*			
*Chlamydomonas acidophilla*	β-carotene	Cu^2+^ 0.1 g/L	120% increase	–	[103]
*Coccomyxa onubensis*		Fe^2+^			[104]
	Lutein	0.5 mM	~33% increase	35% increase	
	Zeaxanthin	0.5 mM	~93% increase	35% increase	
	β-carotene	0.5 mM	~35% increase	35% increase	
*Dunaliella salina*	β-carotene	Fe^2+^ 0→450 µM ^Ac^	7-fold increase	4-fold decrease	[105]
*Nostoc minutum*		As(V)			[34]
	Chlorophyll a	0→1000 mg/L	75% increase	66% increase	
	Carotenoids	0→1000 mg/L	40% increase	66% increase	
	Allophycocyanin	0→1000 mg/L	24.7% increase	66% increase	
*Anabaena doliolum*		Ni^2+^			[92]
	Chlorophyll a	0→10 µM	~47% increase	35% increase ^24h^	
	C-phycocyanin	0→0.1 µM	4.35-fold increase	9% decrease ^96h^	
*Dunaliella salina*	Carotenoids	Cu^2+^1 µM→20 µM	131% increase	>50% decrease	[244]
	Chlorophyll		62% increase		
*Dunaliella tertiolecta*	Carotenoids		133% increase		
	Chlorophyll		152% increase		
*Pseudokirchneriella subcapitata*	Chlorophyll a	Cu^2+^0.5→60 µg/L	10.3-fold increase	Decrease (20% in growth rate and 72% in biomass)	[248]
	Chlorophyll b		15.4-fold increase		
	Carotenoids		4.1-fold increase		
*Scenedesmus obliquus*	Chlorophyll	VO_3_^−^ 0→20 µg/L	100% increase	34% increase	[37]
*Chlorella fusca*	Lutein	VO_3_^−^ 0→20 µg/L ^SFeC^	18% increase	–	[266]
	β-carotene		400% increase		
	Zeaxanthin		130% increase		
*Chlorella fusca*	Lutein	VO_3_^−^ 0→20 µg/L ^FeDC^	17% increase	–	[266]
	β-carotene		200% increase		
	Zeaxanthin		40% increase		
*Haematococcus lacustris*	Carotenoids	VO_4_^3−^0→1.25 mM	125% increase ^2DE^	45% decrease ^2DE^	[39]
*Haematococcus lacustris*	Carotenoids	VO_4_^3−^ 0→1.25 mM	No increase ^4DE^	40% decrease ^4DE^	[39]
		*Lipids*			
*Chlorella minutissima*	Lipids	Cd^2+^ 0→0.4 mM	~94% increase	~12% increase	[115]
*Euglena gracilis*	Lipids	Cr^6+^ 0→1.3 µM ^40%,1^	44% increase ^40%,1^	IC_50_ for 3.2 µM ^1^	[116]
*Euglena gracilis*	Lipids	Cr^6+^ 0→9.84 µM ^40%,2^	28.5% increase ^40%,2^	IC_50_ for 24.6 µM ^2^	[116]
*Euglena gracilis*	Lipids	Cr^6+^ 0→36.16 µM ^40%,3^	100% increase ^40%,3^	IC_50_ for 90.4 µM ^3^	[116]
*Euglena gracilis*	Lipids	Cr^6+^ 0→48.2 µM ^40%,4^	10% increase ^40%,4^	IC_50_ for 120.5 µM ^4^	[116]
*Chlorella vulgaris*	Lipids	TiO_2_-NPs 0→0.1 g/L	10% increase	No change	[74]
*Arthrospira maxima*	Lipids	nZVI-Nanofer 25 0→5.1 mg/L	21% increase	15% increase	[57]
*Desmodesmus subspicatus*	Lipids	nZVI-Nanofer 25 0→5.1 mg/L	58% increase	73% increase	[57]
*Parachlorella kessleri*	Lipids	nZVI-Nanofer 25 0→5.1 mg/L	17% increase	41% increase	[57]
*Nannochloropsis limnetica*	Eicosapentaenoic acid C20:5	nZVI-Nanofer 25 0→5.1 mg/L	58 % increase	19% increase	[57]
*Trachydiscus minutus*	Eicosapentaenoic acid C20:5	nZVI-Nanofer 25 0→5.1 mg/L	34% increase	31% increase	[57]
*Scenedesmus obliquus*	Lipids	(As, Cd, Co, Cr, Cu, Hg, Ni, Pb, Se, Zn) as a mixture	61% increase ^1x^	12% increase ^1x^	[255]
*Neochloris sp.*	Oleic acid C18:1	Effluent from textile dyeing industry containing Pb ^Ut^	Neutral lipid accumulation Oleic acid accumulation	–	[256]
*Chlorella vulgaris*	Lipids	Fe^3+^/EDTA0→12 µM	7.25-fold increase	~27% increase	[263]
*Nannochloropsis oculata*	Lipids	Fe^3+^^+EDTA^ 3.16→18.96 mg/L	22% increase in production	–	[264]
		*Exopolymers*			
*Lyngbya putealis*		Cu		13% decrease	[131]
	Exopolysaccharides	0→2 mg/L	2.43-fold increase		
	Exoproteins	0→2 mg/L	3.65-fold increase		
*Lyngbya putealis*		Co		21% decrease	[131]
	Exopolysaccharides	0→2 mg/L	2.09-fold increase		
	Exoproteins	0→2 mg/L	2.64-fold increase		
*Thalassiosira weissflogii*	Polysaccharides ^EPF^	Ag ^RENP^	~3.5-fold increase ^NL^ if: Ag 0.03→0.11 nM	50% decrease ^NL^ if: Ag 0.01 nM	[132]
*Thalassiosira weissflogii*	Polysaccharides ^EPF^	Ag ^RENP^	~6-fold increase ^NE^ if: Ag0.01→6.14 pM	50% decrease ^NE^ if: Ag 2.16 pM	[132]
*Thalassiosira pseudonana*	Proteins ^EPF^	Cd ^RENP^ 0→0.05 nM	50% increase ^CM,^^NE^	No change ^NE^	[133]
*Thalassiosira pseudonana*	Carbohydrates ^EPF^	Cd ^RENP^ 0→0.05 nM	2-fold increase ^CM,^^NE^	No change ^NE^	[133]
*Cylindrotheca fusiformis*	Exopolysaccharides	Cu^2+^ 0→0.5 mg/L	100% increase ^RC^	57% decrease	[245]
		*Phytohormones*			
*Chlorella vulgaris*	Indole-acetic acid	Cd			[154]
		0→10^−4^ M	~147% increase ^Ct^	~35% decrease ^Ct^	
		0→10^−4^ M ^+B^	3.6-fold increase ^Ct^	~8% decrease ^Ct^	
*Chlorella vulgaris*	Zeatin	Pb			[154]
		0→10^−4^ M	~35% increase ^Ct^	~40% decrease ^Ct^	
		0→10^−4^ M ^+B^	~85% increase ^Ct^	~16% decrease ^Ct^	
*Chlorella vulgaris*	Abscisic acid	Cu			[154]
		0→10^−4^ M	~45% increase^Ct^	~45% decrease ^Ct^	
		0→10^−4^ M ^+B^	~65% increase^Ct^	~24% decrease ^Ct^	
		*Hydrogen*			
*Chlamydomonas reinhardtii*	H_2_	16% leachate medium containing: (Cr, Mn, Fe, Co, Ni, Cu, Mo, Cd, Pb)	~37% increase	~50% increase	[257]
*Anabaena variabilis*	H_2_	VO_3_^−^ 0→0.023 mg/L ^M^	5.5-fold increase	Delayed ^FSC^ No change in growth ^PCT^	[265]
		*Other products*			
*Dunaliella tertiolecta*	Phenolics	Cu^2+^ 0→0.79 µM	40% increase ^RC^	34% decrease	[246]
*Chlorella vulgaris*	Chlorophyll a	Cd^2+^ 0→0.1 µmol/L	~4–fold increase	~65% decrease	[247]
	Protein		~5–fold increase		
	Lipids		~3–fold increase		
*Chlorella pyrenoidosa*	Proline Total Amino Acids	Cr^6+^ 0→5 mg/L	240% increase 66% increase	60% decrease	[63]
*Botryococcus braunii*	Hydrocarbons	Modifications of culture media composition	27% increase after: Fe and Mn uplift + Mo decrease + Ni addition (1.73 µM)	34% increase after: Fe and Mn decrease + Mo uplift + Ni addition (3.38 µM)	[262]

*^Info^*, product synthesis expressed on various basis (cell content, dry weight, release from cells, concentration in the culture, productivity); ^Ac^, with 67.5 mM acetate; ^24h^, a 24h cultivation time; ^96h^, a 96h cultivation time; ^SFeC^, standard Fe concentration; ^FeDC^, Fe deficient conditions; ^2DE^, increase in cells after a 2-day exposure and compared to control cells at the same cultivation time; ^4DE^, increase in cells after a 4-day exposure and compared to control cells at the same cultivation time; ^40%^, concentration that constitutes 40% of a concentration necessary to obtain IC_50_; ^1^, a UTEX strain cultivated in Buetow medium; ^2^, a MAT strain cultivated in Buetow medium; ^3^, a UTEX strain cultivated in C&M medium; ^4^, a MAT strain cultivated in C&M medium; ^1x^, for a lowest metal mixture tested; ^Ut^, Pb was partially utilized by strain; ^+EDTA^, a six fold increase in EDTA concentration also suggested; ^EPF^, from Extracellular Polymeric Fraction; ^RENP^, released from Engineered Nanoparticles; ^NL^, nitrogen limited medium; ^NE^, nutrient enriched medium; ^CM^, in cultivation media; ^RC^, the release from cells; ^+B^, plus brassinolide 10^−8^ M; ^Ct^, when compared to control without heavy metal and brassinolide; ^M^, composition and concentration of other micro/macro nutrients also changed; ^FSC^, during the first stage of cultivation; ^PCT^, in prolonged cultivation time.

## 6. Summary

Metal exposure can be an interesting method to induce, in microalgae cells, the synthesis of target products such as pigments, lipids, peptides, exopolymers, phytohormones, arsenoorganics and nanoparticles. However, stimulation of target compound production in microalgae depends on many factors such as metal type and concentration or metal combination leading to synergistic effects, specificity of strain and cultivation parameters, and growth media composition, which all taken together determines the outcome of microalga response towards metal stress. Moreover, microalgae cultivation under stress conditions can stimulate production of target compounds, but usually at the expense of decreased growth rates, that diminishes overall productivity of metal exposed microalgae systems. The exception are resistant strains isolated from metal contaminated environments. A combination of metal removal from contaminated wastewaters, with metal-induced product biosynthesis, can be applied. Moreover, metal-containing wastewaters could also serve as a replenishment for microalgae growth in nutrient-deficient media. Suitable dosages of metals in relation to selected microalgae strain and adjusted growth conditions is key to develop efficient metal-exposed microalgal production systems.
